# Molecular characterization of class 1, 2 and 3 integrons in clinical multi-drug resistant *Klebsiella pneumoniae* isolates

**DOI:** 10.1186/s13756-019-0509-3

**Published:** 2019-03-29

**Authors:** Farzaneh Firoozeh, Zeinab Mahluji, Ahmad Khorshidi, Mohammad Zibaei

**Affiliations:** 1 Department of Microbiology, School of Medicine, Alborz University of Medical Sciences, Karaj, Iran; 2 Department of Microbiology, School of Medicine, Kashan University of Medical Sciences, Kashan, Iran; 3 Evidence-based Phytotherapy & Complementary Medicine Research Center, Alborz University of Medical Sciences, Karaj, Iran; 4 Department of Parasitology and Mycology, School of Medicine, Alborz University of Medical Sciences, P.O. Box: 3149779453, Karaj, Iran

**Keywords:** *Klebsiella pneumoniae*, Multidrug resistance, Integrons class 1, Integrons class 2, Integrons class 3, Cassette contents

## Abstract

**Background:**

The aim of this study was to characterize class 1,2 and 3 integrons in clinical MDR *Klebsiella pneumoniae* isolates in Kashan, Iran.

**Methods:**

One hundred-eighty one *Klebsiella pneumoniae* were recovered from clinical specimens during November 2013 to October 2014. Antimicrobial susceptibility patterns were determined by disk diffusion method according to the Clinical and Laboratory Standards Institute (CLSI) guidelines for detection of MDR strains. Of the 181 *Klebsiella pneumoniae,* 146 (80.7%) of isolates were isolated from nosocomial infected patients and 150 (82.9%) identified as MDR isolates. The PCR amplification was used to show presence of class 1, 2 and 3 integrons among MDR strains. The PCR method and sequencing were used for evaluation of cassette content of integrons.

**Results:**

Of the MDR *K. pneumoniae* isolates, 150 (100%) and 55 (36.7%) carried *intI1* and *intI2* genes, respectively. None of the MDR *Klebsiella pneumoniae* isolates carried class 3 integrons. Amplification of conserved segment (CS) of class 1 and class 2 integrons revealed 10 different arrays including: No. cassette; *dfrA5*, *dfrA30*; *aadA2*; *aadA2*, *dfrA12*; *dfrA17*, *aadA5*, *aadA4*; *dfrA5*, *dfrA30*, *aadA2*; *dfrA5*, *dfrA30*, *aadA2*, *dfrA12, dfrA5*, *dfrA30*, *dfrA17*, *aadA5*, *aadA4*; *aadA2*, *aadA2*, *dfrA12*; *dfrA5*, *dfrA30*, *aadA2*, *aadA2*, *dfrA12* and 4 arrays including: No. cassette; *aadA1*; *dfrA1-sat1*; *aadA1*, *dfrA1-sat1*, respectively.

**Conclusions:**

The finding of present study revealed a high prevalence of integrons especially class 1 among MDR *K. pneumoniae* isolates from nosocomial infections in Kashan, which led to rapid extension of MDR strains.

## Background

*Klebsiella pneumoniae (K. pneumoniae)* is one of the most important causes of infection especially among hospitalized patients [1]. Multi-drug resistant (MDR) *K. pneumoniae* isolates are becoming increasingly prevalent in the clinical and nosocomial environments and is raised as a major threat in treatment of nosocomial infections [2]. Different mechanisms and factors involved in the development and spread of antibiotic resistance in bacterial strains. Among them acquisition of resistance genes especially via mobile genetic elements is considered as the main factor in the wide distribution of antimicrobial resistance [3]. Integrons which are one of the kind mobile genetic elements presumed to be involved in the dissemination of these MDR strains [4]. Integrons are considered powerful mobile genetic elements that are located on plasmids, transposons and pathogenicity islands which facilitate their transferring among different bacteria. According to reports available, integrons have a wide distribution among clinically isolated bacteria; also, their mobility has become a major problem in antibiotic resistance in clinical specimens [5]. Till now, five classes of integrons have been described based on the nucleotide sequence of the integrase gene [6].

Class 1 integrons are the most prevalent and have been frequently reported in clinical isolates of gram negative bacteria including *K. pneumoniae* [7]. The structure of class 1 integrons is consisted of two conserved regions, including 3′ conserved segment (3′ CS) and 5′ conserved segment (5′ CS), as well as internal gene cassettes that encode antimicrobial resistance genes [7]. Class 2 integrons found sometimes and class 3 integrons are rarely documented in *K. pneumoniae* [3]. Up to now, more than 130 different cassettes which confer resistance against a wide range of antibiotics including all ß- lactams, all aminoglycosides, quinolones, fluoroquinolones, macrolides, and many other antibiotics classes have been detected [5]. Integrons carrying diverse cassette arrays have been identified in different studies in Europe and Asia [5]. Class 1 integrons carries variety of resistance gene cassettes, and most of them contain *aadA* gene which confer resistance to streptomycin- spectinomycin. The wide spread distribution of class 1 integrons harbouring different alleles of the *aadA* gene has been documented [5]. Also, the *dfrA* cassette arrays, encoding resistance to trimethoprim, are frequently detected in both class 1, and 2 integrons [8]. Although several studies have documented the prevalence of integrons in MDR *K. pneumoniae* isolated from clinical specimens in Iran [9, 10], there is little information regarding the association between class 2 and 3 integrons and MDR, also cassettes contents of integrons in *K. pneumoniae* isolates from nosocomial infected patients in our region. Thus, the present study proposed to characterize class 1, 2 and 3 integrons in clinical MDR *K. pneumoniae* isolates in Kashan, Iran.

## Methods

### Bacterial isolates

A total of 181 non-duplicate *K. pneumoniae* isolates from clinical specimens at Shahid Beheshti Hospital in Kashan, Iran, during November 2013 to October 2014 were enrolled in the study. All patients admitted to Shahid Beheshti Hospital in Kashan and diagnosed with infections caused by *K. pneumoniae* during the study were included. While, patients diagnosed with infections due to other bacteria than *K. pneumoniae* during the same time, were excluded. Of the 181 *K. pneumoniae* isolates, 146 (80.7%) were nosocomial and had been occurred after 48 h of hospital admission. The isolates were from both sexes including 78 male and 103 female and were recovered from urine, respiratory tract samples (sputum, bronchoalveolar lavage, tracheal aspirate and nasal discharge), blood, wound, cerebrospinal fluid (CSF) and catheter. Collection of specimens from the lower respiratory tract was done by pulmonologists prior to initiation of antimicrobial therapy. Tracheal aspirate obtained from patients by instillation of 1.5 mL saline (pH 7.0) in to each nostril. After insertion of plastic catheter or tubing contained 2 mL of saline into the nostril, nasopharyngeal secretions were aspirated and collected in sterile containers. Bronchoalveolar lavage (BAL) was obtained using bronchoscope. Also deep cough sputum of other patients with respiratory infections were collected in sterile containers. The collection of CSF was performed by clinicians under aseptic conditions. In addition, in order to obtain urine specimen from patients with urinary tract infection (UTI) and indwelling catheter, sterile syringe was inserted in to catheter at a 45 degree angle, then 20–30 mL of urine was withdrawn and collected in sterile containers.

The isolates were identified as *K. pneumoniae*, after culturing on MacConkey agar (Merck, Germany) media and incubation at 37 °C for 24 h. Characteristic colonies of *K. pneumoniae* were confirmed by standard biochemical tests including TSI (Triple Sugar Iron Agar), Indole, Methyl Red (MR), Voges–Proskauer (VP), and Citrate (IMVIC), also urease and motility tests (Merck, Germany). All *K. pneumoniae* isolates were confirmed as, Indole negative, MR negative, VP positive, Citrate positive, urease positive, and motility negative gram negative coccobacilli [11].

### Antibiotic susceptibility testing

*K. pneumoniae* isolates were tested for susceptibility to antimicrobial agents and identification of MDR strains were done by using disk diffusion method described by the Clinical and Laboratory Standards Institute (CLSI) guidelines [12]. The antibiotics were selected according to CLSI standard and previous studies in this field as follows: ampicillin (30 μg), amoxicillin/clavulanic acid (20/10 μg), aztreonam (30 μg), cephalothin (30 μg), cefotaxime (30 μg), ceftazidime (30 μg), cefoxitin (30 μg), cefteriaxon (30 μg), imipenem (10 μg), gentamicin (10 μg), nalidixic acid (30 μg) and ciprofloxacin (5 μg) (Mast Companies, UK). The *E. coli* strain ATCC 25922 was used as a control. Results were interpreted according CLSI and the manufacturer protocols (Mast, UK) and each *K. pneumoniae* isolate which showed resistance to more than three antibiotic classes was identified as MDR [13].

### Genomic DNA extraction

DNA of each MDR *K. pneumoniae* isolates was extracted by boiling method. The overnight cultures of *K. pneumoniae* strains in LB broth were suspended in 250 μL of sterile deionized water and incubated at 100 °C for 10 min. After centrifugation at 10,000 g for 5 min, the supernatant were used as a template DNA and stored at − 20 °C until use [14].

### Detection and characterization of class 1, 2 and 3 integrons

The presence of class 1, 2 and 3 integrons in MDR *K. pneumoniae* were investigated by amplification of integrase genes including *intI1*, *intI2*, and *intI3* specific primers (Table 1). The PCR reactions were prepared in a total volume of 25 μL and amplification was performed in a thermaocycler (Eppendorf master cycler®, MA) as follows: 5 min at 94 °C; 35 cycles of 1 min at 94 °C, 1 min at 55 °C,30 s at 72 °C; 10 min at 72 °C for detection of *intI1* gene and 5 min at 94 °C; 32 cycles of 1 min at 94 °C, 1 min at 60 °C,2 min at 72 °C; 10 min at 72 °C for detection of *intI2*, and *intI3* genes [15–17]. Reaction mixtures without a DNA template used as negative control. The amplified products were electrophoresed on 1.2% agarose gel and after staining with ethidium bromide (0.5 mg/ml) visualized in gel document system (Biorad, UK).

**Table 1:** Primers used for PCR amplification for detection of class 1, 2 and 3 integrons

Gene	Primer	Sequence (5′-3′)	Amplification Product (bp)	Reference
*intI1*	IntI1-F	TCTCGGGTAACATCAAGG	254	[11]
	IntI-R	AGGAGATCCGAAGACCTC		
*intI2*	IntI2-F	CACGGATATGCGACAAAAAGG	788	[12]
	IntI2-R	TGTAGCAAACGAGTGACGAAATG		
*intI3*	IntI3-F	AGTGGGTGGCGAATGAGTG	600	[13]
	IntI3-R	TGTTCTTGTATCGGCAGGTG		
5′CS	5′CS-F	GGCATCCAAGCAGCAAG	Variable	[14]
3′CS	3′CS-R	AAGCAGACTTGACCTGA		
*attI2*	attI2-F	GACGGCATGCACGATTTGTA	Variable	[14]
*orfX*	orfX-R	GATGCCATCGCAAGTACGAG		

### Detection and characterization of integrons internal variable region genes

All integron–positive MDR *K. pneumoniae* isolates were tested for the presence of internal cassettes genes by CS-PCR using 3′CS and 5′CS primers (Table 1). The following conditions was used for CS-PCR reaction: initial denaturation at 94 °C for 5 min, followed by 35 cycles of denaturation at 94 °C for 1 min, annealing at 58 °C for 1 min, and extension at 72 °C for 2 min, with final extension at 72 °C for 10 min [18]. After electrophoresis on 1.2% agarose gel and staining, the PCR products were visualized.

### DNA sequencing and integrons gene cassettes analysis

The purified PCR products were sequenced by the ABI Capillary System and Sanger’s method (Macrogen Research, Seoul, Korea) using 10 pmol of specific primers. The sequences were analyzed by Chromas Pro version1.7.5 Technelysium as well as online BLAST software (http://www.ncbi.nlm. nih.gov/BLAST/). Sequences of CS-PCR products revealed the integrons cassettes contents.

## Results

This study was conducted on 181 patients with age ranged between 1 and 97 (mean 50.36 ± 3.80 years). The *K. pneumoniae* were isolated from clinical specimens including: urine 124 (68.5%), wound 6 (3.3%), blood 5 (2.8%), respiratory tract samples 43 (23.8%) including (6 sputum, 2 bronchoalveolar lavage, 34 tracheal aspirates and 1 nasal discharge), CSF 1 (0.6%) and catheter 2 (1.1%).

**Fig. 1:**
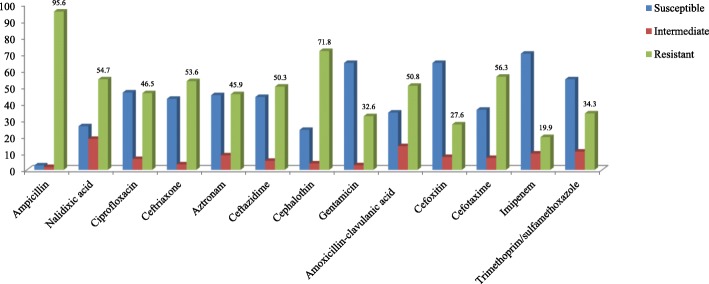
The antibiotic resistant patterns of *K. pneumoniae* isolates measured by disk diffusion method (*N* = 181)

The antibiotic susceptibility patterns by disk diffusion are shown in (Fig. 1). The highest resistance was obtained to ampicillin, cephalothin, cefotaxime and cefteriaxon. One hundred- fifty (82.9%) identified as MDR isolates, and showed resistance to more than three antimicrobial families. Class 1 and 2 integrons were detected in 150 (100%) and 55(36.7%) of MDR *K. pneumoniae* isolates, which showed to carry *intI1* and *intI2* genes respectively. The *intI3* gene was not identified among 150 MDR *K. pneumoniae* isolates and class 3 integrons were not founded in any MDR *K. pneumoniae* isolates.

Sequencing analysis for *intI*-positive strains revealed that the cassette arrays of class 1 integron were including 10 different arrays groups from A-J (Table 2), consist of (708 bp, 1002 bp, 1500 bp and 1610 bp integrons) and identified gene cassettes were as follows: (no cassette; *dfrA5*, *dfrA30*; *aadA2*; *aadA2*, *dfrA12*; *dfrA17*, *aadA5*, *aadA4*; *dfrA5*, *dfrA30*, *aadA2*; *dfrA5*, *dfrA30*, *aadA2*, *dfrA12*; *dfrA5*, *dfrA30*, *dfrA17*, *aadA5*, *aadA4*; *aadA2*, *aadA2*, *dfrA12*; *dfrA5*, *dfrA30*, *aadA2*, *aadA2*, *dfrA12*) Whereas, 4 different cassette arrays groups from a-d (Table 3) consist of (1000 bp and 1500 bp integrons) were detected among 55 MDR *K. pneumoniae* isolates which carried class 2 integrons, and identified gene cassettes were as follows: (no cassette; *aadA1*; *dfrA1-sat1*; *aadA1*, *dfrA1-sat1*).

**Table 2:** Sources, numbers, sizes, inserted cassettes and groups of cassettes arrays of integrons, among integron class 1-positive *K. pneumoniae* isolates

Source	Isolate (N) (%)	*intI1* (No = 150)	Cs_1_ (No = 132)	NO. of Integrons Class1	Integron Sizes(s) (bp)	Inserted Cassette(s)	Groups of Cassettes Arrays of Integrons Class 1
Urine	14 (9.3)	+	–	–	–	NO cassette	A
	25 (16.6)	+	+	2	708	*dfrA5*, *dfrA30*	B
	3 (2.0)	+	+	1	1002	*aadA2*	C
	13 (8.7)	+	+	2	1500	*aadA2*, *dfrA12*	D
	10 (6.7)	+	+	3	1610	*dfrA17*, *aadA5*, *aadA4*	E
	7 (4.6)	+	+	3	708,1002	*dfrA5*, *dfrA30*, *aadA2*	F
	13 (8.7)	+	+	4	708,1500	*dfrA5*, *dfrA30*, *aadA2*, *dfrA12*	G
	2 (1.3)	+	+	5	708, 1610	*dfrA5*, *dfrA30*, *dfrA17*, *aadA5*, *aadA4*	H
	4 (2.6)	+	+	3	1002,1500	*aadA2*, *aadA2*, *dfrA12*	I
	8 (5.3)	+	+	5	708,1002, 1500	*dfrA5*, *dfrA30*, *aadA2*, *aadA2*, *dfrA12*	J
Respiratory	3 (2.0)	+	+		–	NO cassette	A
	15 (10)	+	+	2	708	*dfrA5*, *dfrA30*	B
	1 (0.7)	+	+	1	1002	*aadA2*	C
	10 (6.7)	+	+	2	1500	*aadA2*, *dfrA12*	D
	6 (4.0)	+	+	3	1610	*dfrA17*, *aadA5*, *aadA4*	E
	2 (1.3)	+	+	3	708,1002	*dfrA5*, *dfrA30*, *aadA2*	F
	1 (0.7)	+	+	4	708,1500	*dfrA5*, *dfrA30*, *aadA2*, *dfrA12*	G
	1 (0.7)	+	+	3	1002,1500	*aadA2*, *aadA2*, *dfrA12*	I
	1 (0.7)	+	+	5	708,1002, 1500	*dfrA5*, *dfrA30*, *aadA2*, *aadA2*, *dfrA12*	J
Blood	1 (0.7)	+	+	3	1610	*dfrA17*, *aadA5*, *aadA4*	E
	1 (0.7)	+	+	3	708,1002	*dfrA5*, *dfrA30*, *aadA2*	F
Wound	3 (2.0)	+	+	2	708	*dfrA5*, *dfrA30*	B
	3 (2.0)	+	+	3	1610	*dfrA17*, *aadA5*, *aadA4*	E
CSF	1 (0.7)	+	+		–	NO cassette	A
Catheter	2 (1.3)	+	+	2	1500	*aadA2*, *dfrA12*	D

**Table 3:** Sources, numbers, sizes, inserted cassettes and groups of cassettes arrays of integrons, among integron class 2-positive *K. pneumoniae* isolates

Source	Isolate N (%)	*intI2* (No = 55)	Cs_2_ (No = 37)	NO. of Integrons Class 2	Integron Sizes(s) (bp)	Inserted Cassette(s)	Groups of Cassettes Arrays of Integrons Class 2
Urine	12 (21.9)	+	–	–	–	NO cassette	a
	7 (12.7)	+	+	1	1000	*aadA1*	b
	6 (10.9)	+	+	2	1500	*dfrA1-sat1*	c
	7 (12.7)	+	+	3	1000,1500	*aadA1*, *dfrA1-sat1*	d
Respiratory	3 (5.5)	+	–	–	–	NO cassette	a
	3 (5.5)	+	+	1	1000	*aadA1*	b
	6 (10.9)	+	+	2	1500	*dfrA1-sat1*	c
	4 (7.3)	+	+	3	1000,1500	*aadA1*, *dfrA1-sat1*	d
Blood	1 (1.8)	+	–	–	–	NO cassette	a
	1 (1.8)	+	+	3	1000,1500	*aadA1*, *dfrA1-sat1*	d
Wound	2 (3.6)	+	–	–	–	NO cassette	a
	1 (1.8)	+	+	1	1000	*aadA1*	b
	2 (3.6)	+	+	3	1000,1500	*aadA1*, *dfrA1-sat1*	d

The most common cassettes were 708 bp, which were detected in 43 (28.6%) isolates with class 1 integrons (Table 2), whereas among class 2 integrons, the most frequent cassettes were 1000–1500 bp, which were identified among 14 (25.5%) of them (Table 3). Twenty- three (15.3%) of class 1 integron positive *K. pneumoniae* strains carried more than three gene cassettes simultaneously consisting of array of 708,1500 bp; 708, 1610 bp; 708, 1002, 1500 bp.

## Discussion

MDR *K. pneumoniae* has become an important challenge in treatment of nosocomial infections worldwide [19]. It has been documented that mobile genetic elements, such as integrons, play an important role in the dissemination of MDR- *K. pneumoniae* isolates [20]. In this study we have characterized the class 1, 2 and 3 integrons in clinical MDR *K. pneumoniae* isolates in Iran. All MDR *K. pneumoniae* that we investigated were positive for class 1 integrons. The intense association between the presence of class 1 integrons and occurrence of MDR among gram negative bacteria has been documented [21, 22]. In Li et al. [21] study, the class 1 integron positive isolates in comparison with class 1 integron negative isolates showed resistance to a much higher number of drugs [21]. High frequency of integron positive MDR- *K. pneumoniae* has been reported from other studies [22, 23]. High Prevalence of integrons among MDR strains, could be due to that integrons confer a selection advantage to strains that live in environments such a hospitals where selective pressures created by overuse of antibiotics. We found that among the integron class 1 positive strains, 10 amplicon were identified. The most prevalent arrays observed among class 1 integrons were 708 bp and gene cassettes identified were *dfrA5* and *dfrA30* which encode dihydrofolate reductases enzymes. Also other variants of *dfrA* genes including *dfrA12* and *dfrA17* were identified in relatively high frequency among our integron class 1 positive *K. pneumonia* strains*.* The studies show that the most prevalent integron cassette- associated genes are those encode dihydrofolate reductases and aminoglycoside modifying enzymes [21, 24–26]. Salimizand et al. [9] reported *dfrA17* variant in *Klebsiella* species. The cassette arrays of class 1 integron in *intI1*-positive *K. pneumoniae* strains in China were included *dfrA17*, *dfrA12, dfrA1*, *dfrA25*, *dfrA27*, genes [19, 21]. The *dfrA17* and *dfrA12* have been identified among gram negative bacteria that carried class 1 integrons in USA [27], which shows these variants are common among cassettes of class 1 integrons in the world. In other studies in Iran other variants of *dfrA* genes including *dfrA7*, *dfrA1 dfrA25*, *dfrA5* and *dfrA12* have been documented among *Salmonella* serotypes and Enteropathogenic *Escherichia coli* (EPEC) isolates respectively [24, 28–30]. To our knowledge this is the first report of detecting *dfrA5, dfrA12* and *dfrA30* variants among *K. pneumoniae* in Iran. Also detection of *dfrA5, dfrA12* variants in other species could be due to interspecies gene transfer. The second most prevalent cassette in our study was *aadA2* which were identified in 67 intI1-positive *K. pneumoniae* strains. The frequency of presence of other variants of *aadA* genes including *aadA4* and *aadA5* were also relatively high in studied intI1-positive *K. pneumoniae* strains. Till now, 18 different variants of the *aadA* genes which encode resistance to streptomycin- spectinomycin have been identified on gene cassettes of class 1 integrons among gram-negative bacteria [26]. The presence of *aadA2* is shown in *Salmonella* serotypes and EPEC with low prevalence in Iran [24, 30]. In studies conducted in Taiwan, *aadA* genes variants including *aadA1*, *aadA2* and *aadA4* have been reported in majority of MDR *Acinetobacter baumannii* isolates and integrons class 1 carrying *aadA2* variant was the most frequently found cassette array in clinical isolates of *K. pneumoniae* [31, 32]. In a study in Iran *aadA5* gene have been identified among integron cassettes of MDR *Klebsiella* spp. [9]*.* According to data in the literature this is the first report of *aadA2* variant in *K. pneumoniae* in Iran and presence of this gene in different studies among cassettes arrays of class 1 integrons shows that this gene may be the first cassette to be captured by an integron. Another interesting founding was that relatively high frequency of class 1 integron positive *K. pneumoniae* strains carried more than three gene cassettes simultaneously. This result in correlation with other studies in different geographical areas shows the high diversity of integrons among *K. pneumoniae* isolates in our regions [7, 32, 33]. The frequency of class 2 integrons in our MDR *K. pneumoniae* isolates was 36.7% which is higher than that reported by Ahangarzadeh Rezaee et al. [10] in northwest of Iran. The frequencies of 14, 4.8, and 10.4% have been reported in *Acinetobacter baumannii*, EPEC and *Escherichia coli* isolates from humans and animals in Iran and different parts of the world respectively [34–37]. In study of cassettes contents of class 2 integrons in MDR *K. pneumoniae* isolates for the first time in Iran, we found that the most prevalent cassettes arrays observed among class 2 integrons were *aadA1* and *dfrA1-sat1* which confer resistance to streptomycin and spectinomycin, trimethoprim and streptothricin respectively. In other studied these cassettes arrays are frequently detected among class 2 integrons [18, 38]. In a study conducted by Eftekhari et al. [39], these cassettes were identified in class 2 integrons in *Shigella* spp. isolated from patients in Iran.

The high frequency of *aadA1* and *dfrA1-sat1* genes which has been documented in different reports, shows the stability of these gene cassettes among class 2 integrons especially in gram negative bacteria, also the identification of these gene cassettes among other genus of Enterobacteriaceae family is evidence of interspecies transition of class 2 integrons. We found an apparent association between the presence of *aad* and *drf* gene cassette arrays and phenotypic resistance to the corresponding antibiotics in our *int*-positive strains. On the other hand, despite the observation of high phenotypic resistance to many other antibiotics such as ampicillin, no cassette carrying the gene for resistance to these antibiotics was obtained. This could be due to the fact that the resistance genes to these antibiotics are located outside the integrons. Of the all integron class 1 and 2 positive *K. pneumoniae* strains, 12.0 and 37.2% isolates harboured empty integrons and did not carried any gene cassettes. Empty integrons have been documented by other reports [18, 39] indicate the potential of these isolates to capture resistance gene cassettes and change to strains with multiple resistance determinants especially in hospitals environments due to antibiotic selective pressures.

## Conclusion

Over all, the results of in this study revealed a high prevalence of integrons especially class 1 among MDR *K. pneumoniae* isolates from nosocomial infections in Kashan, which led to rapid extension of MDR strains.
